# Association of the interleukin-22 genetic polymorphisms with ulcerative colitis

**DOI:** 10.1186/s13000-014-0183-y

**Published:** 2014-10-09

**Authors:** Hong Gang Chi, Xue Bao Zheng, Zhu Guo Wu, Shi Xue Dai, Zheng Wan, Ying Zou

**Affiliations:** Department of Traditional Chinese Medicine, The Second Clinical Medical College, Guangdong Medical College, 1 Xincheng Road, Songshan LakeSci.&Tech, Industry Park, Dongguan, Guangdong 523808 China; The Second Clinical Medical College, Guangdong Medical College, Dongguan, 523808 China; Emergency Department of Nanfang Hospital, Southern Medical University, Guangzhou, 510515 China; Sino-American Cancer Research Institute, Guangdong Medical College, Dongguan, 523808 China

**Keywords:** Interleukin-22, Gene polymorphism, Ulcerative colitis, Case–control study

## Abstract

**Background:**

Interleukin-22 (*IL-22*) is a member of the *IL-10* family of anti-inflammatory cytokines that mediates epithelial immunity. *IL-22* expression was found to be increased in patients with ulcerative colitis (UC). Whether genetic polymorphisms of *IL-22* also influence UC risk is still unknown. The purpose of this study was to investigate the association between the *IL-22* gene polymorphisms (−429 C/T, +1046 T/A and +1995 A/C) and the risk of UC in Chinese Han patients.

**Methods:**

This hospital-based case–control study comprised 180 patients with UC and 180 age- and gender-matched controls. Genotypes of 3 common polymorphisms of the *IL-22* gene were determined by fluorogenic 5′ exonuclease assays (TaqMan).

**Results:**

Patients with UC had a significantly higher frequency of *IL-22* −429 TT genotype [odds ratio (OR) =2.43, 95% confidence interval (CI) = 1.35, 4.37; *P* = 0.003] and −429 T allele (OR =1.54, 95% CI = 1.14, 2.07; *P* = 0.004) than controls. The findings are still emphatic by the Bonferroni correction. The *IL-22* +1046 T/A and *IL-22* +1995 A/C gene polymorphisms were not associated with a risk of UC. When stratifying by clinical type, location and disease severity of UC, no significant differences were found in any groups.

**Conclusion:**

This is the first study to provide evidence for an association of *IL-22* −429 C/T gene polymorphisms with UC risk. Additional well-designed large studies were required for the validation of our results.

**Virtual Slides:**

The virtual slide(s) for this article can be found here: http://www.diagnosticpathology.diagnomx.eu/vs/13000_2014_183

## Background

Ulcerative colitis (UC), as the most common form of inflammatory bowel disease (IBD), is a form of colitis that includes characteristic ulcers, or open sores [1]. Ulcerative colitis has an incidence of 1 to 20 cases per 100,000 individuals per year, and a prevalence of 8 to 246 per 100,000 individuals [2]. Patients with UC have an increased risk of developing colorectal cancer [3]. The pathogenesis of UC is not well defined, but it is proposed that genetic and environmental factors result in an aberrant immune response to a subset of commensal enteric bacteria [4,5]. Recent genome-wide association (GWA) studies and meta-analyses have identified many single-nucleotide polymorphisms (SNPs) associated with UC [6,7].

Interleukin-22 (*IL-22*) is a member of the *IL-10* family of anti-inflammatory cytokines that mediates epithelial immunity [8]. *IL-22* is synthesized by different cell types including T- and NK-cells, and has been reported to mediate the crosstalk between inflammatory cells and keratinocytes [9-11]. *IL-22* expression was induced in several human inflammatory conditions, including IBD [12,13]. *IL-22* expression was found to be increased in patients with UC [14]. Several SNPs have previously been identified in the *IL-22* gene [15-22].

Whether genetic polymorphisms of *IL-22* also influence UC risk is still unknown. The aim of this study was to investigate the association between the *IL-22* gene polymorphisms (−429 C/T, +1046 T/A and +1995 A/C) and the risk of UC in Chinese Han patients.

## Methods

### Study subjects

This hospital-based case–control study comprised 180 patients with UC and 180 age- and gender-matched controls during the years 2009 to 2014. Diagnosis of UC was based on clinical, radiological, endoscopic and histological examinations [23]. To confirm the diagnosis, two physicians reviewed the hospital records and validated each case. The location of disease was defined according to the Montreal classification (ulcerative proctitis, left-sided colitis, and extensive colitis) [24]. The severity of UC was determined according to Truelove and Witts criteria (mild colitis, moderate colitis, and severe colitis) [25]. According to their clinical courses, UC cases were classified into one episode, relapsing and continuous phenotypes [26]. The control group was composed of age- and gender-matched subjects who had undergone an endoscopic examinations in the same recruitment period as the UC patients, without evidence of UC. In addition, similar to the cases the controls were all required to be born in China to native Chinese Han parents. Informed consent was obtained from all subjects after a full explanation of the project according to the Declaration of Helsinki, and the specimen collecting procedures were approved by the Institutional Ethical Committee of Guangdong Medical College.

### Genotyping

Whole blood (3–6 ml) was obtained by venepuncture using standard EDTA collection tubes. DNA was extracted using the QIAGEN Gentra Puregene blood kit (QIAGEN Inc., Valencia, CA, USA). Genotypes of 3 common polymorphisms of the *IL-22* gene were determined by fluorogenic 5′ exonuclease assays (TaqMan). Primer and probe sequences for 5′-exonuclease assays for *IL-22* polymorphisms were listed in Table 1. The polymerase chain reaction (PCR) was performed in a Primus 96 plus thermal cycler using a total volume of 5 μl containing 2.5 μl of Universal-MasterMix, 0.125 μl 40x Assay-by- Design mix, 0.375 μl H_2_O and 2 μl DNA. Reactions were overlaid with 15 μl of mineral oil. Cycling parameters were: 10 min at 94°C for primary denaturation, followed by 40 cycles of 20 s at 92°C and 1 min at 60°C. Fluorescence was measured in a lambda Fluoro 320 Plus plate reader (MWG Biotech AG, Germany).

**Table 1:** **Primer and probe sequences for 5′-exonuclease assays for** ***IL-22*** **polymorphisms**

**SNPs**	**rs2227485**	**rs1182844**	**rs1179246**
Exchange	−429 C/T	+1046 T/A	+1995 A/C
Forward Primer	AAAATGAGTCCGTGACCAAAATGC	CCACCTATGAGACTTCCCTATCAGT	GAAAAAGCCTTCCTGCCTAATGG
Reverse Primer	ACACAATTGTTTTGTCTTAGTAGAGTTCAGAT	CACTAAAGGAAAAGGAAAGCTGTGTTT	GGTGCTGCCTAAAGGTCAGA
Wildtype-Probe	FAM-CTCCTATAGTGACTGAGTAA-NFQ	VIC-AAACTTACTAGTAGGTATGACTC-NFQ	VIC-TGAACAGAGTTATCTGCCTC-NFQ
Mutant-Probe	VIC-CTCCTATAGTGGCTGAGTAA-NFQ	FAM-CTTACTAGTAGGAATGACTC-NFQ	FAM-AACAGAGTTAGCTGCCTC-NFQ

Abbreviations: *SNP* single nucleotide polymorphisms.

### Statistical analysis

STATA program version 11.0 (StataCorp LP, TX) was used for statistical analyses. Continuous variables were analysed by *t*-test and presented as mean ± standard deviation (SD). Categorical variables are presented as percentages and were compared by chi-squared test. Odds ratio (OR) and 95% confidence intervals (CI) were determined by logistic regression analysis. For a new work, we also use the Bonferroni correction for total number of independent comparisons. The Hardy-Weinberg equilibrium was tested for goodness-of-fit chi-square test with one degree of freedom to compare the observed genotype frequencies among the subjects with the expected genotype frequencies. The *P*-value less than 0.05 was considered statistically significant.

## Results

### Characteristics of participants

Demographic and clinical characteristics of study participants were showed in Table 2. No significant differences were found between the UC cases and controls in age, sex, body mass index (BMI), smoking status and family history of inflammatory bowel disease (IBD) (Table 2). The genotype were in agreement with the Hardy-Weinberg equilibrium.

**Table 2:** **Demographic and clinical characteristics of study participants**

	**UC**	**Controls**	***P***
Number of subjects	180	180	
Sex (Male/Female)	95/85	98/82	0.75
Age (years, mean ± SD)	39.7 ± 12.5	40.2 ± 13.1	0.71
BMI (kg/m^2^, mean ± SD)	20.2 ± 3.7	19.8 ± 3.5	0.29
Smoking status (Ever/Never)	79/101	75/105	0.67
Family history of IBD (Positive/Negative)	14/166	11/169	0.54
Clinical type			
One episode	16		
Relapsing	70		
Continuous	94		
Location			
Proctitis (E1)	73		
Left side (E2)	64		
Extensive (E3)	43		
Disease severity			
Mild	76		
Moderate	95		
Severe	9		

Abbreviations: *UC* ulcerative colitis, *BMI* body mass index, *IBD* inflammatory bowel disease.

### ***IL-22*** −429C/T polymorphisms and UC

Patients with UC had a significantly higher frequency of *IL-22* −429 TT genotype (OR =2.43, 95% CI = 1.35, 4.37; *P* = 0.003) and −429 T allele (OR =1.54, 95% CI = 1.14, 2.07; *P* = 0.004) than controls (Table 3). The findings are still emphatic by the Bonferroni correction. When stratifying by clinical type, location and disease severity of UC, no significant differences were found in any groups (Table 4).

**Table 3:** **Genotype and allele frequencies of** ***IL-22*** **gene polymorphisms among ulcerative colitis cases and healthy controls**

**Genotypes**	**UC (n = 150)**	**Controls (n = 150)**	**OR (95% CI)**	***P***
−429 CC	57(31.7)	68(37.8)	1.00(Reference)	
−429 CT	70(38.9)	86(47.8)	0.97(0.61,1.56)	0.90
−429 TT	53(29.4)	26(14.4)	2.43(1.35,4.37)	0.003
−429 C allele frequency	184(51.1)	222(61.7)	1.00(Reference)	
−429 T allele frequency	176(48.9)	138(38.3)	1.54(1.14,2.07)	0.004
+1046 TT	88(48.9)	94(52.2)	1.00(Reference)	
+1046 TA	63(35.0)	61(33.9)	1.10(0.70,1.74)	0.67
+1046 AA	29(16.1)	25(13.9)	1.24(0.67,2.28)	0.49
+1046 T allele frequency	239(66.4)	249(69.2)	1.00(Reference)	
+1046 A allele frequency	121(33.6)	111(30.8)	1.14(0.83,1.55)	0.43
+1995 AA	76(42.2)	81(45.0)	1.00(Reference)	
+1995 AC	65(36.1)	63(35.0)	1.10(0.69,1.75)	0.69
+1995 CC	39(21.7)	36(20.0)	1.16(0.67,2.00)	0.61
+1995 A allele frequency	217(60.3)	225(62.5)	1.00(Reference)	
+1995 C allele frequency	143(39.7)	135(37.5)	1.10(0.81,1.48)	0.54

Abbreviations: *UC* ulcerative colitis, *OR* odds ratio, *CI* confidence interval.

**Table 4:** **Stratification analysis of** ***IL-22*** **−429 C/T polymorphisms in ulcerative colitis cases**

	**Cases (n = 180)**	**CC**			**CT**			**TT**		
		**n (%)**	**OR (95% CI)**	***P***	**n (%)**	**OR (95% CI)**	***P***	**n (%)**	**OR (95% CI)**	***P***
Clinical type	180	57(31.7)	1(Reference)		70(38.9)	1(Reference)		53(29.4)	1(Reference)	
One episode	16	5(31.2)	0.99(0.35,2.81)	0.98	7(43.8)	1.13(0.44,2.85)	0.80	4(25.0)	0.85(0.27,2.65)	0.78
Relapsing	70	24(34.3)	1.08(0.62,1.88)	0.78	26(37.1)	0.96(0.56,1.62)	0.87	20(28.6)	0.97(0.54,1.74)	0.92
Continuous	94	28(29.8)	0.94(0.56,1.58)	0.82	37(39.4)	1.01(0.63,1.62)	0.96	29(30.8)	1.05(0.63,1.76)	0.86
Location	180	57(31.7)	1(Reference)		70(38.9)	1(Reference)		53(29.4)	1(Reference)	
Proctitis (E1)	73	24(32.9)	1.04(0.60,1.80)	0.89	27(37.0)	0.95(0.57,1.60)	0.85	22(30.1)	1.02(0.58,1.80)	0.94
Left side (E2)	64	20(31.3)	0.99(0.55,1.77)	0.97	26(40.6)	1.05(0.61,1.78)	0.87	18(28.1)	0.96(0.52,1.75)	0.88
Extensive (E3)	43	13(30.2)	0.96(0.48,1.90)	0.89	17(39.6)	1.02(0.54,1.90)	0.96	13(30.2)	1.03(0.51,2.05)	0.94
Disease severity	180	57(31.7)	1(Reference)		70(38.9)	1(Reference)		53(29.4)	1(Reference)	
Mild	76	25(32.9)	1.04(0.61,1.79)	0.89	28(36.8)	0.95(0.57,1.58)	0.84	23(30.3)	1.03(0.59,1.80)	0.92
Moderate	95	29(30.5)	0.96(0.58,1.61)	0.89	38(40.0)	1.03(0.65,1.64)	0.91	28(29.5)	1.00(0.59,1.68)	0.99
Severe	9	3(33.3)	1.05(0.28,4.02)	0.94	4(44.5)	1.14(0.34,3.83)	0.83	2(22.2)	0.76(0.16,3.60)	0.72

Abbreviations: *OR* odds ratio, *CI* confidence interval.

### ***IL-22*** +1046 T/A polymorphisms and UC

The *IL-22* +1046 T/A gene polymorphisms were not associated with a risk of UC (Table 3). When stratifying by clinical type, location and disease severity of UC, no significant differences were found in any groups (Table 5).

**Table 5:** **Stratification analysis of** ***IL-22*** **+ 1046 T/A polymorphisms in ulcerative colitis cases**

	**Cases (n = 180)**	**TT**			**TA**			**AA**		
		**n (%)**	**OR (95% CI)**	***P***	**n (%)**	**OR (95% CI)**	***P***	**n (%)**	**OR (95% CI)**	***P***
Clinical type	180	88(48.9)	1(Reference)		63(35.0)	1(Reference)		29(16.1)	1(Reference)	
One episode	16	7(43.7)	0.89(0.35,2.55)	0.81	6(37.6)	1.07(0.40,2.86)	0.89	3(18.7)	1.16(0.32,4.25)	0.82
Relapsing	70	33(47.1)	0.96(0.59,1.57)	0.88	26(37.2)	1.06(0.62,1.81)	0.83	11(15.7)	0.98(0.46,2.06)	0.95
Continuous	94	48(51.1)	1.04(0.68,1.61)	0.84	31(33.0)	0.94(0.57,1.55)	0.82	15(15.9)	0.99(0.51,1.94)	0.98
Location	180	88(48.9)	1(Reference)		63(35.0)	1(Reference)		29(16.1)	1(Reference)	
Proctitis (E1)	73	37(50.7)	1.04(0.65,1.66)	0.88	25(34.2)	0.98(0.57,1.67)	0.94	11(15.1)	0.94(0.44,1.97)	0.86
Left side (E2)	64	33(51.6)	1.06(0.65,1.72)	0.83	21(32.8)	0.94(0.53,1.66)	0.82	10(15.6)	0.97(0.45,2.10)	0.94
Extensive (E3)	43	18(41.9)	0.86(0.47,1.57)	0.62	17(39.5)	1.13(0.60,2.12)	0.70	8(18.6)	1.16(0.49,2.70)	0.74
Disease severity	180	88(48.9)	1(Reference)		63(35.0)	1(Reference)		29(16.1)	1(Reference)	
Mild	76	35(46.1)	0.94(0.59,1.51)	0.81	28(36.8)	1.05(0.63,1.77)	0.85	13(17.1)	1.06(0.52,2.15)	0.87
Moderate	95	48(50.5)	1.03(0.67,1.59)	0.88	32(33.7)	0.96(0.59,1.58)	0.88	15(15.8)	0.98(0.50,1.92)	0.95
Severe	9	5(55.6)	1.14(0.37,3.49)	0.82	3(33.3)	0.95(0.25,3.63)	0.94	1(11.1)	0.69(0.08,5.65)	0.73

Abbreviations: *OR* odds ratio, *CI* confidence interval.

### ***IL-22*** +1995 A/C polymorphisms and UC

The *IL-22* +1995 A/C gene polymorphisms were not associated with a risk of UC (Table 3). When stratifying by clinical type, location and disease severity of UC, no significant differences were found in any groups (Table 6).

**Table 6:** **Stratification analysis of** ***IL-22*** **+1995 A/C polymorphisms in ulcerative colitis cases**

	**Cases (n = 180)**	**AA**			**AC**			**CC**		
		**n (%)**	**OR (95% CI)**	***P***	**n (%)**	**OR (95% CI)**	***P***	**n (%)**	**OR (95% CI)**	***P***
Clinical type	180	76(42.2)	1(Reference)		65(36.1)	1(Reference)		39(21.7)	1(Reference)	
One episode	16	7(43.8)	1.04(0.41,2.62)	0.94	6(37.5)	1.04(0.39,2.77)	0.94	3(18.7)	0.87(0.24,3.12)	0.83
Relapsing	70	29(41.4)	0.98(0.59,1.63)	0.94	25(35.7)	0.99(0.58,1.69)	0.97	16(22.9)	1.06(0.55,2.01)	0.87
Continuous	94	40(42.6)	1.01(0.64,1.59)	0.97	34(36.2)	1.00(0.62,1.63)	0.99	20(21.2)	0.98(0.54,1.78)	0.95
Location	180	76(42.2)	1(Reference)		65(36.1)	1(Reference)		39(21.7)	1(Reference)	
Proctitis (E1)	73	31(42.5)	1.01(0.61,1.66)	0.98	26(35.6)	0.99(0.58,1.68)	0.96	16(21.9)	1.01(0.53,1.92)	0.97
Left side (E2)	64	26(40.6)	0.96(0.57,1.63)	0.89	24(37.5)	1.04(0.60,1.80)	0.89	14(21.9)	1.01(0.52,1.98)	0.98
Extensive (E3)	43	19(44.2)	1.05(0.57,1.91)	0.88	15(34.9)	0.97(0.50,1.86)	0.92	9(20.9)	0.97(0.44,2.15)	0.93
Disease severity	180	76(42.2)	1(Reference)		65(36.1)	1(Reference)		39(21.7)	1(Reference)	
Mild	76	33(43.4)	1.03(0.63,1.68)	0.91	27(35.5)	0.98(0.58,1.66)	0.95	16(21.1)	0.97(0.51,1.84)	0.93
Moderate	95	39(41.1)	0.97(0.61,1.54)	0.91	35(36.8)	1.02(0.63,1.65)	0.94	21(22.1)	1.02(0.57,1.83)	0.95
Severe	9	4(44.5)	1.05(0.32,3.52)	0.93	3(33.3)	0.92(0.24,3.52)	0.91	2(22.2)	1.03(0.21,4.93)	0.98

## Discussion

Recently, many studies have identified many SNPs associated with UC. A case–control study in 139 and 176 patients with UC and controls found that polymorphisms in *CD14* −159 C/T and *TLR4* −299 A/G significantly affected mCD14 and mTLR4 expression levels and also increased susceptibility to UC [27]. A case–control study in a Korean population suggested that the −1920 G > A polymorphism in *IFITM1* may be associated with susceptibility to UC (*P* = 0.002) [28]. A case–control study in a group of 198 Mexican Mestizo patients with UC and 698 ethnically matched healthy unrelated individuals with no family history of UC suggested that the GG genotypes of the *IL-20* polymorphisms (rs2981573 and rs2232360) might have an important role in the development of UC (*P* = 0.017) in the Mexican population [29]. A case–control study in 56 patients with UC and 50 healthy controls suggested that the *IL-10* −819 CC was a candidate genotype for both IBS (*P* = 0.047) and UC (*P* = 0.007) in Japanese [30]. A case–control study suggested that the −1850 G/C polymorphism in the embryonic ectoderm development (*EED*) gene might be associated with the susceptibility to UC (*P* = 0.018) by the change of the *EED* expression level [31]. A meta-analysis of seventeen studies with 18,308 cases and 20,406 controls indicated that the *PTPN2* polymorphism (rs2542151) was associated with increased UC risk [32]. A meta-analysis of nine studies indicated that the vitamin D receptor (*VDR*) polymorphisms were associated with increased UC risk [33]. A meta-analysis suggested that the migration inhibitory factor (*MIF*) gene −173 G/C polymorphism contributed to the susceptibility of UC [34]. A case–control study included 422 very-early-onset IBD subjects and 480 healthy subjects suggested that *IL-10R* polymorphisms were associated with very-early-onset ulcerative colitis (*P* = 0.0002) [35]. A case–control study included 171 UC and 213 healthy controls found that polymorphisms in *XRCC1* Arg399Gln and *APE1* Asp148Glu significantly increased the rate of apoptosis and risk of UC (*P* = 0.0007) [36]. A case–control study in a group of 200 Mexican patients with UC and 248 ethnically matched unrelated healthy controls suggested that *IL-1 RN* and *IL-1B* polymorphisms were associated with the genetic susceptibility to develop UC and might be associated with the presence of steroid-dependence in UC patients (*P* = 0.019) [37]. A case–control study in a group of 200 Mexican Mestizo patients with UC and 698 healthy unrelated individuals with no family history of UC suggested that *IL-19* polymorphisms (rs2243188 and rs2243193) might have a protective role in the development of UC (*P* = 0.018 and *P* = 0.006, respectively) in Mexican individuals [38].

*IL-22* expression was involved in several human inflammatory conditions and autoimmune diseases. A study included 631 HCV patients found that *IL-22* polymorphisms were involved in the progression of persistent hepatitis C virus infection [15]. A case–control study in 206 cases and 196 controls suggested that Polymorphisms in the *IL-22* receptor alpha-1 gene were associated with severe chronic rhinosinusitis (*P* = 0.0014) [16]. A case–control study in 194 patients and 287 normal controls suggested that polymorphism of *IL-22* receptor alpha-1 was associated with the development of childhood IgA nephropathy (*P* = 0.002) [22]. *IL-22* deficiency may contribute to the pathogenesis of certain chronic disorders as postulated in this paper for acne inversa [39]. A study included 94 patients with COPD, 23 healthy smokers, and 22 healthy control non-smokers found that the increased sputum *IL-22* might also play important roles in the pathogenesis of chronic obstructive pulmonary disease (*P*< 0.01) [40]. A case–control study indicated that there was an increased expression level of *IL-22* and *IL-23* in patients with peri-implantitis (*P*< 0.05) [41]. A study in 18 cases and 21 controls suggested that increased expression of *IL-22* was associated with disease activity in Behcet’s disease [42]. *IL-22* has been reported to be involved in systemic sclerosis lesions [43]. A study included allergic asthma (n = 18), controlled asthma (n = 17) and healthy controls (n = 12) found that *IL-22* might be involved in the pathogenesis of allergic asthma in human and the level of *IL-22* might have some relationship with the severity of the disease (*P*< 0.05) [44].

Our results should be taken with caution for some limitations. First of all, the numbers of subjects included in this study were small, and may not have been sufficient to reveal the associations between the *IL-22* gene polymorphisms (−429 C/T, +1046 T/A and +1995 A/C) and the risk of UC. Secondly, our investigation was not based on genome wide screening, but UC was induced by multiple genes and environmental factors, which were not explored in the present study. Thirdly, the participants in our research are only from Han Chinese ethnic group. It would be interesting to conduct similar studies in different populations for comparison. Finally, this is a hospital based case control study, so the selection bias cannot be avoidable and the subjects may not be representative of the general population.

## Conclusions

In conclusion, this study provides evidence for an association of *IL-22* −429 C/T gene polymorphisms with UC risk. To the best of our knowledge this is the first study to provide evidence about the role of *IL-22* polymorphisms in the development of UC. Additional well-designed large-scale multicenter studies were required for the validation of our results.
